# Mental health status according to the dual-factor model in Swedish adolescents: A cross sectional study highlighting associations with stress, resilience, social status and gender

**DOI:** 10.1371/journal.pone.0299225

**Published:** 2024-03-01

**Authors:** Veronica Hermann, Fredrik Söderqvist, Ann-Christin Karlsson, Anna Sarkadi, Natalie Durbeej

**Affiliations:** 1 Child Health And Parenting (CHAP), Department of Public Health and Caring Sciences, Uppsala University, Uppsala, Sweden; 2 University Health Care Research Centre, Faculty of Medicine and Health, Örebro University, Örebro Sweden; 3 Center for Clinical Research, Uppsala University, County Hospital, Västerås, Sweden; 4 Department of Public Health and Caring Sciences, Uppsala University, Uppsala, Sweden; USM Advanced Medical and Dental Institute: Universiti Sains Malaysia Institut Perubatan dan Pengigian Termaju, MALAYSIA

## Abstract

In this cross-sectional study, we aimed to I) investigate the dual-factor model of mental health by forming and describing four participant groups and II) examine associations between mental health status and background factors, school-related factors, stress, and resilience among adolescents in a community population in Sweden. Data were collected through a survey completed by 2,208 students in lower and upper secondary school on the Swedish island of Gotland. After missing data were removed, a total of 1,833 participants were included in the study. The survey included the Mental Health Continuum Short Form (MHC-SF) for the assessment of mental well-being and the Strengths and Difficulties Questionnaire (SDQ) for the assessment of mental health problems. These two measures were combined into a dual-factor model, forming four mental health status subgroups: *Vulnerable* (47.5%), *Complete mental health* (36.2%), *Troubled* (13.9%), and *Symptomatic but content* (2.5%). Associations between these groups were explored regarding background factors, school-related factors, stress, and resilience through chi-squared tests and logistic regressions. Girls (OR: 1.88) and participants with high stress levels (OR: 2.23) had elevated odds for *Vulnerable mental* health status, whereas higher resilience (OR: 0.87) and subjective social status in school (OR: 0.76) were factors associated with reduced odds for this mental health status classification. Female gender (OR: 5.02) was also associated with *Troubled* mental health status. Similarly, a high level of stress (ORs: 4.08 and 11.36) was associated with S*ymptomatic but content* and *Troubled* mental health status, and participants with higher levels of resilience had decreased odds for being classified into these groups (ORs: 0.88 and 0.81). The findings highlight the importance of interventions to increase resilience, reduce stress, and address stereotypic gender norms as well as social status hierarchies to support adolescents’ mental health.

## Introduction

Adolescents’ mental health is a highlighted public health issue [1]. Approximately 13–19% of adolescents worldwide suffer from some kind of mental health problem [1–4], and research suggests an increased prevalence of mental health problems in adolescents [1, 4–8].

In order to provide adequate assessment and interventions, adolescents’ mental health status needs to be fully understood [9]. Overall, the mental health field has a long tradition of focusing merely on the concept of mental health problems [10]. The latter can be divided into a) mental difficulties, i.e., symptoms not reaching international diagnostic criteria for psychiatric conditions, albeit causing suffering and difficulties to maintain daily life and b) psychiatric conditions in line with international diagnostic criteria [11].

Mental well-being is a concept that is traditionally described as the absence of mental health problems [10, 12]. However, currently, mental wellbeing and mental health problems are described as two different continua, albeit occurring in close relation to each other. Keyes [13] defined the mental well-being continuum as encompassing three aspects of well-being: 1) emotional well-being, 2) psychological functioning, and 3) social functioning. Based on the presence of these three different parts, an individual’s mental well-being can be described as flourishing (i.e., high mental well-being) or languishing (i.e., low mental well-being), or somewhere in between. The three core parts of mental well-being are also visible in the World Health Organization’s definition of mental health [12] stated as “A state of well-being in which the individual realizes his or her own abilities, can cope with the normal stresses of life, can work productively and fruitfully, and is able to make a contribution to his or her community” [14].

Since mental well-being can be defined as a distinct dimension of a person’s mental health, rather than just the absence of being mentally unwell, merely focusing on mental health problems in order to assess mental health status has been suggested as being too narrow of an approach [10, 12]. Consequently, the dual-factor model of mental health was proposed to assess total mental health status, involving the presence and absence of both mental wellbeing and mental health problems [10].

According to this model, four logically defined mental health status groups, based on responses to instruments measuring well-being and mental health problems, can be formed [10]. The groups can be described as 1) individuals with *Complete mental health*, including high levels of mental well-being and low levels of mental health problems, 2) *Symptomatic but content* individuals, with high levels of mental wellbeing and high levels of mental health problems, 3) *Vulnerable* individuals with low levels of mental well-being and low levels of mental health problems, and 4) *Troubled* individuals with low mental well-being and high levels of mental health problem [15].

Overall, the dual-factor model is applicable to adolescents and has been supported by adolescents themselves [16]. Studies have reported quite some variation in adolescents who experience *Complete mental health*, with estimates from 19 to 78% [9, 15, 17–24]. Further, in these studies, 5 to 50% of the participants were classified as having *Vulnerable* mental health status, and 4 to 36% as having *Symptomatic but content* status. Finally, 3 to 25% were classified into the *Troubled* mental health status group. Hence, the proportion of participants classified in the different mental health status groups differs widely between studies. Apart from different settings and ages of participants, these variations might be explained by different measures used and whether established score-based or relative distribution-based cut-offs have been used to assess well-being and mental health problems [9, 17–28].

Research exploring the mental health status of groups involving assessment instruments corresponding to Keyes’ [13, 29] definition of mental well-being is limited as well as requested [21, 23, 27]. A majority of the aforementioned studies on adolescents’ mental health according to the dual-factor model have been conducted in the United States [9, 15, 17–20, 22–24, 26]. To the best of our knowledge, no Swedish studies on this topic have been published, despite the fact that the increase in mental health difficulties among young Swedish people stands out compared to other countries [8, 30]. Thus, research using the dual-factor model might provide a more nuanced exploration of the mental health status of Swedish adolescents and point to potential solutions and support needs.

Research has suggested that the dual-factor model can provide information that is otherwise impossible to acquire within the traditional single model of mental health, i.e., mental health assessment based only on the presence or absence of mental health problems [9, 27]. Assessment of mental well-being alongside an assessment of mental health problems, in the dual-factor model, acknowledges adolescents in the *Symptomatic but content group* and the *Vulnerable mental health status group*, who might be overlooked using traditional mental health assessments [9, 10, 27]. If assessed using traditional methods, adolescents with *Vulnerable* mental health status might not receive mental health interventions, despite having low levels of mental well-being [9, 10, 22]. Hence, descriptions of adolescents in the four mental health status groups are needed to guide decisions on mental health interventions based on both status of mental well-being and mental health problems [10].

Apart from genetic factors, several other factors are associated with adolescents’ mental health, either affecting mental well-being or mental health problems or both [1, 31]. Mental well-being in adolescents, for instance, has been associated with gender norms, school achievement, socioeconomic situation of the family, country of birth, and resilience [1, 31–35]. It is defined as a “concept referring to positive adaptation, or the ability to maintain or regain mental health, despite experiencing adversity” [36]. Some studies report that boys have better mental well-being than girls [1, 35] and that younger adolescents have better mental well-being than older adolescents [37].

Mental health problems in adolescents have been associated with migration experiences, low family socioeconomic status (SES), low social status in school, unsatisfactory educational achievements, and high levels of stress, e.g., related to stressful life events, academic achievements, and interpersonal stress [1, 22, 33, 38]. Self-reported mental health problems have been proposed to be more common in girls than boys [4]. In addition, according to The United Nations Children’s Fund (UNICEF) [1], specific psychiatric conditions, such as anxiety and depressive disorders, are more common among girls than among boys, while boys are more likely to have conduct disorder and ADHD (Attention Deficit Hyperactivity Disorder). Overall, mental health problems are more common among older than younger adolescents [1, 4]. The risk of mental health problems may also be increased by the lack of coping strategies but reduced by high levels of resilience [22, 32]. In addition, an association has been reported between high mental well-being and decreased mental health problems in youth [37].

Some of the above-mentioned factors have previously been explored in relation to the dual-factor model among adolescents [9, 15, 17, 21–24]. The results indicate that the four mental health status groups differ when it comes to SES, grades, school attendance, and experience of stressful life events. However, more research on the associations between mental health status, according to the dual-factor model, and associated factors could be useful when planning for interventions to improve adolescents’ mental health. For instance, group differences might highlight the need for delivery of different interventions to each group.

To summarize, there is a research gap on the prevalence of adolescents’ mental health status according to the dual-factor model. Moreover, there is a need for more studies exploring the associated factors of being classified into the four mental health status groups. This could be considered particularly important, given the increase in mental health problems and low mental well-being in adolescents [1, 4–8, 35]. Hence, this study aimed to investigate the dual-factor model in forming and describing four participant groups relating to mental health among adolescents in a community population in Sweden. In addition, the study aimed to examine the association between mental health status subgroups and background factors, school-related factors, stress, and resilience in this population.

## Methods

### Study design

We used a cross-sectional study design with data collection through a survey of Swedish students in lower and upper secondary school. Data were gathered from the Life and Health of Youth (LHY), an online survey administered to students in grades 7–12, in the Gotland region, Sweden.

### Setting and population

All twelve lower and upper secondary schools (grades 9–12) on the Swedish island of Gotland (with approximately 61,000 inhabitants) were recruited and participated in the study between April and June 2021. Among a total of 3,541 students attending these schools, 2,208 students responded to the survey, with a response rate of 62%. To minimize the risk of unreliable responses, 24 students, with two standard deviations above or under the mean on items measuring height and length, were excluded, as is the routine for these types of surveys in Sweden [39]. A total of 59 participants with other gender identity than boy or girl, or who were uncertain of their gender identity, were also excluded for the purposes of this study. In addition, we excluded another 292 participants with missing data on certain variables of interest: gender, country of birth, parents/legal guardians occupation, grade, given the grade F or no grade in any subjects, having skipped school, feeling stressed, and the measures for assessing resilience and mental health problems Thus, a total of 1,833 students comprised the final dataset.

### Measures and variables

#### Mental well-being

Well-being was assessed using the Mental Health Continuum Short Form (MHC-SF) [40], including 14 items that can be divided into three subscales considering emotional well-being, psychological and social functioning, corresponding to mental well-being symptoms, as described by Keyes [13, 29]. The items are rated depending on whether symptoms have been evident during the past month, on a 6-point scale from 0 (never) to 5 (every day), with a total score ranging from 0 to 70. A higher score is equivalent to a higher level of well-being.

Respondents are defined as having “flourishing” mental health if they report frequent positive experiences (“every day” or “almost every day”) on at least one of the three items assessing emotional well-being and at least six of the eleven items assessing positive functioning.

Furthermore, respondents are defined as having “languishing” mental health if they seldom report positive experiences (“never” or “once or twice”) according to at least one of the three items assessing emotional well-being and at least six of the 11 items assessing positive functioning. Individuals who are neither “languishing” nor “flourishing” are defined as having “moderate positive mental health.” Individuals in the categories “languishing” and “moderate positive mental health” can also be collapsed into a category with “moderate to low mental health” [41].

The MHC-SF has demonstrated sufficient psychometric properties in both international and Swedish research on adolescents [42–44]. In the current study, the internal consistency reliability for the total scale estimated by Cronbach’s alpha was 0.91.

#### Mental health problems

Mental health problems were assessed through the Swedish self-report version of the Strengths and Difficulties Questionnaire (SDQ) [45, 46]. The instrument comprises 25 items that can be divided into five subscales: emotional symptoms, conduct problems, hyperactivity, peer problems, and pro-social behavior. A total difficulties score can be calculated by adding up the subscales (except for the pro-social behavior subscale), ranging between 0 and 40. A higher score corresponds to a higher level of problems. A dichotomous variable for scoring above or below the cut-off for the total SDQ score, indicating possible mental health problems or no mental health problems can be computed. Given that there are no Swedish norms available, we used the score ≥ 20 as the cutoff for defining mental health problems. This was based on the 90^th^ percentile from a population-based UK survey [47]. The SDQ has established psychometric properties in adolescents aged 11–17-years-old [48, 49] and has also been found to be applicable for adolescents 17–19-years-old [50, 51]. In the current study, Cronbach’s Alpha for the SDQ total scale was 0.74.

#### Mental health status according to the dual-factor model

In order to form the four mental health status groups, as proposed in the dual-factor model, we combined the participants’ results regarding “flourishing” or “moderate to low’” mental wellbeing according to the MHC-SF [40] and the dichotomous variable for scoring above or below the cut-off for the total SDQ score for indicating possible mental health problems or no mental health problems [45]. The groups were defined as: *Complete mental health*, with flourishing mental well-being and no mental health problems; *Symptomatic but content*, with flourishing mental well-being and mental health problems; *Vulnerable*, with moderate to low mental well-being and no mental health problems; and *Troubled*, with moderate to low mental well-being and mental health problems.

#### Background factors, school-related factors, stress, and resilience

A number of items from the LHY were used in order to measure factors associated with mental health (Table 1). They included gender, birth country, parents/legal guardians’ occupation, given the grade F or no grade in any subjects, truancy, feeling stressed, resilience, and subjective social status (SSS) in school. SSS in school was measured by the youth version of the MacArthur Scale of Subjective Status [52]. This instrument has previously been used to measure the socioeconomic status among adolescents, with good reliability. Resilience was measured by the Swedish translation of the Child and Youth Resilience Measure-12, CYRM-12 [53, 54]. In the current study, Cronbach’s Alpha for the CYRM-12 was 0.75.

**Table 1:** Survey questions and independent variables in the analysis.

Questions in the survey	Independent variables in the analysis	
What is your gender identity?	**Gender**	
	*Boy*	
	*Girl*	
Where were you born?	**Birth country**	
	*Sweden*	
	*Other country*: based on responses “Somewhere else in Europe” or ‘Somewhere else in the world”.	
What is the main occupation of your parent/guardian A?	**Parents/legal guardians unemployed or on long-term sick-leave**	
What is the main occupation of your parent/guardian B?	*No one*: based on the responses “Working”, “Studying”, “Sick-leave <6 months”, “Other occupation”, or “Don’t know”.	
	*At least one*: based on responses “Unemployed” or “Long-term sickleave/ long-term sick-leave, or retired due to disability”.	
What grade are you in?	**Grade**	
	*Grade 7–9*, *lower secondary school*	
	*Grade 10–12*, *upper secondary school*	
Have you been given the grade F or no grade in any subjects?	**Given the grade F or no grade in any subjects**	
	*In no subject*: based on the response “No, not in any subjects”, i.e., approved school results according to the Swedish school system.	
	*In at least one subject*: based on the response “Yes, in 1–2 subjects”,	
	“Yes, in 3–4 subjects”, or “Yes, in 5 subjects or more”, i.e., not approved school results according to the Swedish school system.	
Do you usually skip school?	**Truancy**	
	*Never*: based on the response “No”.	
	*At some point during the semester or more often*: based on the response “Yes, some time per semester”, “Yes, some time per month”, “Yes, 2–3 times per month”, “Yes, some time per week”, or “Yes, several times per week”.	
Do you feel stressed?	**Feeling stressed**	
	*No or to some extent*: based on the responses “No” or “To some extent”.	
	*Yes*, *to a considerable extent*: based on the responses “Fairly much” or “Very much”	
CYRM-12^a^ includes 12 questions about resilience which cover individual, relational, contextual, and cultural aspects.	**Resilience score**	
	The responses are graded from one to five, giving a maximum score of 60 points. A higher score represents a higher degree of resilience.	
Where would you place yourself on this ladder?	**Subjective social status (SSS) in school**
Assume that the ladder (with ten steps) is a way of picturing your school. At the top of the ladder are the students with the most respect, who everyone wants to hang around with, and who have the highest standing. At the bottom of the ladder are the students who no one respects, no one wants to hang around with, and who have the lowest standing.	Each step on the ladder corresponds to a score between one and ten. The lowest step is equal to score one and the highest step is equal to score ten. Thus, a higher score corresponds to higher SSS in school.

^a^ Child and Youth Resilience Measure-12

### Procedure

Students completed the LHY online during class hours, in a test-like situation. Students were given oral and written information about the purpose of the study and told that participation was voluntary. Students in grades 7–8 answered the survey questions anonymously, while students in grades 9–12, who had turned 15-years-old, had the option of answering either anonymously or with their social security number and their data being pseudonymized. The purpose of gathering this information was to be able to follow up on students later on through registries, using their personal identification numbers. Legal guardians were given written information about the study and the possibility of contacting the school if they did not want their child to participate in the study. Students absent on the day of the survey did not participate in the study.

### Statistical analyses

Frequencies, percentages, means, and standard deviations were used for descriptive purposes.

The Spearman’s rank correlation coefficient was used to explore the relation between the MHC-SF total score and the SDQ total difficulties score. Chi-square analyses and Fisher’s Exact test (when appropriate) were used to compare the participant groups regarding the categorical data. To compare participant groups regarding continuous data, One-way ANOVA with Tukey Post hoc test was used. Prior to computing the parametric tests, the Skewness and kurtosis values were computed to examine the normal distribution of the data. The SSS in school score, the CYRM-12 total score, the MHC-SF, and SDQ total difficulties scores were regarded as, approximatively, normally distributed with the skewness values ranging from 0.84 to 0.33 and kurtosis values ranging from -0.06 to 1.49.

Effect sizes on significant differences between the groups were explored by Cohen’s h for categorical variables and Cohen’s d for continuous variables. Single and multiple binary logistic regressions were used to explore the associations between the independent variables (i.e., gender, birth country, grade, given the grade F or no grade in any subjects, truancy, feeling stressed, resilience, and SSS in school) and classification into the mental health status groups. Multiple variable regression was used when the number in each group allowed for it, in order to avoid residual confounding or type II error due to low statistical power. Odds ratios (OR), confidence intervals (CI), and R^2^ values are reported from the regressions. In addition, in the multiple regression, the model fit is reported according to the Hosmer-Lemeshow test.

All independent variables were checked for multicollinearity through the examination of the Variance Inflation Factor (VIF) values. The VIF-values were <10 (range 1.03–1.81), indicating that multicollinearity was not present in the data [55].

The analyses were conducted in SPSS, version 28, with the exception of the Fishers’ Exact test, which was computed in R, version 4.2.1. Bonferroni correction for multiple testing was applied (= 0.05/37); thus, P-values < 0.0014 were considered statistically significant.

### Ethical considerations

Since some of the participants provided identifying information and did not participate anonymously, ethical approval was sought and granted by the Swedish Ethical Review Authority, reg.no.2020-07190. Regardless whether the participants answered anonymously or not, data were only analyzed on a group level. Hence, participants with mental health problems according to the SDQ were not identified or further assessed to confirm these results. However, all participants received written information on where to turn to seek support and treatment if needed.

### Data management

The data were stored digitally at Uppsala University in a way to prevent access from unauthorized persons. Data from participants who partook with their personal identification numbers were pseudonymized, i.e. the data were linked to a serial number and the code list linking the serial and personal numbers was stored separately from the data. During and after the data collection, the first, fourth and fifth author had access to all data (anonymimized and psudonymized) and the fifth author has access to the code list.

## Results

### Population characteristics

Descriptive data for the study are reported in Table 2. The population was equally distributed in terms of gender (boys 49.8%, girls 50.2%). The participants’ age ranged from 12 to > 20 years, where 11 participants were older than 20 years. The mean age of the participants 12–20 years old (not shown in table) was 15.8 years (SD = 1.7). A majority were born in Sweden (93%). A total of 6.1% had at least one parent/legal guardian who was unemployed or on long-term sick-leave or retired due to disability. Approximately one-fifth (20.6%) had been given the grade F in at least one subject, and 29.2% had been truant at some point or more often during the semester. Moreover, 41.9% perceived feeling stressed to a considerable extent. The mean scores of the SSS in school and CYRM-12 were 6.5 (SD = 1.8) and 45.4 (SD = 6.8), respectively.

**Table 2:** Descriptive data for the participating adolescents (n = 1833).

Variables	n (%)
**Gender**	
Girl	920 (50.2)
Boy	913 (49.8)
**Grade**	
7^th^	266 (14.5)
8^th^	391 (21.3)
9th	379 (20.7)
10^th^	274 (14.9)
11^th^	316 (17.2)
12^th^	207 (11.3)
**Birth country**	
Sweden	1699 (92.7)
Other country	134 (7.3)
**Have two parents/legal guardians** ^a^	
Yes, have two parents/legal guardians	1796 (98.0)
No, do not have two parents/legal guardians	37 (2.0)
**Parents/legal guardians unemployed or on long-term sick-leave/retired due to disability**	
No one	1722 (93.9)
At least	111 (6.1)
**Occupation parent/legal guardian A**	
Working	1660 (90.6)
Studying	50 (2.7)
Unemployed	29 (1.6)
Long-term sick-leave/retired due to disability	21 (1.1)
Sick-leave < 6 months	23 (1.3)
Other unspecified occupation	26 (1.4)
Unknown occupation	24 (1.3)
**Occupation parent/legal guardian B**	
Working	1549 (84.5)
Studying	49 (2.7)
Unemployed	39 (2.1)
Long-term sick-leave/retired due to disability	30 (1.6)
Sick-leave < 6 months	36 (2.0)
Other unspecified occupation	43 (2.3)
Unknown occupation	50 (2.7)
Do not have two parents/legal guardians	37 (2.0)
**Given the grade F or no grade in any subject**	
In no subject	1455 (79.4)
In at least one subject	378 (20.6)
**Truancy**	
Never	1297 (70.8)
At some point during the semester or more often	536 (29.2)
**Feeling stressed**	
No or to some extent	1065 (58.1)
Fairly much or very much	768 (41.9)
**Subjective social status (SSS) in school score**b	
1 (lowest)	10 (0.5)
2	41 (2.2)
3	56 (3.1)
4	124 (6.8)
5	236 (12.9)
6	268 (14.6)
7	364 (19.9)
8	349 (19.0)
9	126 (6.9)
10 (highest)	59 (3.2)
**SSS in school score**a Mean (SD)	6.5 (1.8)
**Resilience (CYRM-12) score** Mean (SD)	45.4 (6.8)

^a^ Refers to whether the participants have one or two parents/legal guardians but not whether they live with none, one, or two parents/legal guardians.

^b^ For SSS in school, n = 1633 since 200 of the 1,833 participants did not answer this question.

The mean MHC-SF and SDQ total difficulties scores were 42.7 (SD = 14.1) and 13.6 (SD = 5.9), respectively (Table 3), and there was a negative correlation between the two scales (r = -0.50, p < .001). A total of 38.6% had flourishing mental well-being, whereas 61.4% had moderate to low well-being, according to the MHC-SF. A total of 16.4% had mental health problems, according to the SDQ. Approximately half of the participants were classified as belonging to the *Vulnerable* mental health status group (47.5%), whereas a minority were classified as belonging to the *Symptomatic but content* group (2.5%) (Table 4).

**Table 3:** Mental well-being and mental health problems as measured by the Mental Health Continuum Short Form (MHC-SF) and the Strengths and Difficulties Questionnaire (SDQ) (n = 1833).

Variables	n (%)	Mean (SD)	Median	10^th^	90^th^
				percentile	percentile
**Level of Mental well-being**		42.74 (14.1)^a^	44.0 ^a^	23.0 ^a^	59.0 ^a^
**according to the MHC-SF**					
Flourishing mental well-being	708 (38.6)				
Moderate to low mental well-being	1125 (61.4)				
**Level of mental health problems**		13.6 (5.9)	13.0	6.00	21.6
**according to the SDQ**					
Mental health problems	300 (16.4)				
No mental health problems	1533 (83.6)				

^a^ Since the total MHC-SF scores are summarized only for participants who answered all 12 questions, 208 participants with missing data on at least one of the 12 questions in the MHC-SF were excluded, leaving n = 1625.

**Table 4:** Mental health status groups yielded from the dual-factor model of mental health (n = 1833).

	Level of mental health problems according to the SDQ		*p*
Level of mental well-being according to the MHC-SF	No mental health problems	Mental health problems	
Flourishing mental well-being	*Complete mental health* (C)	*Symptomatic but content* (S)	**<0.001**
	n = 663 (36.2%)	n = 45 (2.5%)	
Moderate to low well-being	*Vulnerable* (V)	*Troubled* (T)	
	n = 870 (47.5%)	n = 255 (13.9)	

### Mental health status group descriptions

#### Complete mental health group

This mental health group was the second largest group (36.2%), with 663 participants who were classified as having flourishing mental well-being according to the MHC-SF but no mental health problems according to the SDQ (Table 4). The proportion of boys (63.2%) was larger compared to the proportion of boys in the *Troubled* group (25.5%), (Cohen’s h 0.78, p <0.001) (Table 5). The current group included relatively few participants who had grade F or no grade in any subjects (15.5%) or who had been truant (20.8%), compared with the *Symptomatic but content* group (51.1% and 64.4%, respectively) and the *Troubled* group (36.9% and 51.0%, respectively) (Cohen’s h 0.50–0.92, p <0.001). The majority (78.1%) reported that they were not particularly stressed (either not stressed or stressed to some extent). This proportion was larger compared with the equivalent proportions in the other mental health status groups (23.9–53.4%) (Cohen’s h: 0.53–1.15, p <0.001). Further, in this group, the participants had the highest mean resilience score, relative to the other three groups (Cohen´s h 0.73–1.39, p <0.001) and a higher mean SSS score compared with the *Vulnerable* and *Troubled* mental health status groups (Cohen’s d 0.62 and 1.10, respectively, p <0.001).

**Table 5:** Background and self-rated health in the mental health status groups (n = 1833). Significant differences between proportions are highlighted in bold.

	Mental health status groups						
	*Complete*	*Symptomatic*	*Vulnerable (V)*	*Troubled (T)*			
	*mental health (C)*	*but content (S)*					
				*n = 255 (13*.*9%)*			
	*n = 663 36*.*2%)*	*n = 45 (2*.*5%)*	*n = 870 (47*.*5%)*				
*Categorical variables*	n (%)	n (%)	n (%)	n (%)	*p*	Column proportions	Cohen’s h
**Gender**							
Girl	244 (36.8)	23 (51.1)	463 (53.2)	190 (74.5)	**<0.001**	T>V>C; T>S	T vs. V = 0.45; T vs. C = 0.78
							V vs. C = 0.33; T vs. S = 0.49
Boy	419 (63.2)	22 (48.9)	407 (46.8)	65 (25.5)		C>V>T; S>T	C vs. V = 0.33; C vs. T = 0.78
							V vs. T = 0.45; S vs. T = 0.49
**Birth country**							
Sweden	622 (93.8)	37 (82.2)	808 (92.9)	232 (91.0)	0.030	-	
Other country	41 (6.2)	8 (17.8)	62 (7.1)	23 (9.0)		-	
**Parents/legal guardians unemployed or on long-term sick-leave/retired due to disability**							
No one	632 (95.3)	40 (88.9)	816 (93.8)	234 (91.8)	0.081	-	
At least one	31 (4.7)	5 (11.1)	54 (6.2)	21 (8.2)		-	
**Grade**							
Grade 7–9, lower secondary school	400 (60.3)	21 (46.7)	459 (52.8)	156 (61.2)	0.005	-	
Grade 10–12, upper secondary school	263 (39.7)	24 (53.3)	411 (47.2)	99 (38.8)		-	
**Given the grade F or no grade in any subjects**							
In no subjects	560 (84.5)	22 (48.9)	712 (81.8)	161 (63.1)	**<0.001**	C,V> S,T	C vs. S = 0.78; C vs. T = 0.50
							V vs. S = 0.71; V vs. T = 0.42
In at least one subject	103 (15.5)	23 (51.1)	158 (18.2)	94 (36.9)		S,T>C,V	S vs. C = 0.78; S vs. V = 0.71
							T vs. C = 0.50; T vs. V = 0.42
**Truancy**							
Never	525 (79.2)	16 (35.6)	631 (72.5)	125 (49.0)	**<0.001**	C>V>S,T	C vs. V = 0.16; C vs. S = 0.92
							C vs. T = 0.64; V vs. S = 0.76; V vs. T = 0.49
At some point during the semester or	138 (20.8)	29 (64.4)	239 (27.5)	130 (51.0)		S,T>V>C	S vs. V = 0.76; S vs. C = 0.92
more often							T vs. V = 0.49; T vs. C = 0.64; V vs. C = 0.16
**Feeling stressed**							
No or to some extent	518 (78.1)	21 (46.7)	465 (53.4)	61 (23.9)	**<0.001**	C>V,S>T	C vs. V = 0.53; C vs. S = 0.66
							C vs. T = 1.15; V vs. T = 0.62; S vs. T = 0.48
Yes, to a considerable extent	145 (21.9)	24 (53.3)	405 (46.6)	194 (76.1)		T>S,V>C	T vs. S = 0.48; T vs. V = 0.62
							T vs. C = 1.15; S vs. C = 0.66; V vs. C = 0.53
***Continuous variables***					***p***	***PostHoc***	**Cohen’s d**
**Resilience (CYRM-12) score**	48.9 (5.6)	42.8 (7.5)	44.6 (6.2)	40.0 (7.1)	**<0.001**	C>V>T; C>S	C vs. V = 0.73; C vs. T = 1.39
Mean (SD)							V vs. T = 0.69; C vs. S = 0.92
**Subjective social status (SSS) in school score** ^a^ **(**Mean (SD)	7.3 (1.5)	6.9 (1.8)	6.3 (1.7)	5.3 (2.1)	**<0.001**	C>V>T; S>T	C vs. V = 0.62; C vs. T = 1.10
							V vs. T = 0.52: S vs. T = 0.82

^a^ Since 200 of the 1,833 participants did not answer this question, n = 1633; group C (n = 588), group S (n = 31), group V (n = 788), group T (n = 226)

#### Symptomatic but content group

This mental health status group was the smallest group (2.5%), with 45 participants who were classified as having flourishing mental well-being according to the MHC-SF, in combination with mental health problems according to the SDQ (Table 4). In this group, just over half (51.1%) had F or no grade in at least one subject, and almost two-thirds (64.4%) reported that they had been truant at least once during the semester (Table 5). These proportions were larger than the equivalent proportions of the *Complete mental health* and the *Vulnerable* groups (Cohen’s h 0.71–0.92, p<0.001). Furthermore, the proportion of participants who perceived feeling stressed to a considerable extent (53.3%) was larger than the equivalent proportions of the *Complete mental health group* (21.9%) and smaller than in the *Troubled* group (76.1%) (Cohen’s h 0.66 and 0.48, respectively, p<0.001). Moreover, the resilience scores in this group were lower than in the *Complete mental health* group (Cohen’s d 0.92, p < 0.001), and the SSS in school scores were higher compared with the *Troubled* group (Cohen’s d 0.82, p <0.001).

#### Vulnerable group

This was the largest group, with nearly half (n = 870) of the participants (47.5%) who were classified as having moderate to low mental well-being according to the MHC-SF, with absence of mental health problems according to the SDQ (Table 4). The majority of the participants did not have F or no grade in any subjects (81.8%) (Table 5). This proportion was larger than in the *Symptomatic but content* group (48.9%) (Cohen’s h 0.71, p <0.001). Furthermore, *vulnerable* participants were more likely not to not to be a truant (72.5%) compared with participants in the S*ymptomatic but content* group (35.6%) and *Troubled* group (49.0%), (Cohen’s h 0.49 respectively, 0.76, p < .001). In the current group, the proportion of participants who perceived feeling stressed to a considerable extent (46.6%) was larger than in the *Complete mental health* group (21.9%) (Cohen’s h 0.53, p <0.001) but smaller than in the *Troubled* group (76.1%) (Cohen’s h 0.62, p <0.001). In addition, resilience and SSS in school scores were lower among the *vulnerable* participants than among the participants in the *Complete mental health* group (Cohen’s d 0.73 and 0.62, respectively) but higher than among the participants of the *Troubled* group (Cohen’s d 0.0.69 and 0.52, respectively).

#### Troubled group

This group comprised 255 participants (13.9%), classified as having moderate to low mental well-being according to the MHC-SF, and concurrent mental health problems according to the SDQ (Table 4). A majority were girls (74.5%), with a larger proportion than the proportion of girls in the *Complete mental health* group (36.8%) and *Symptomatic but content* group (51.1%) (Cohen’s h 0.78, respectively 0.49, p <0.001) (Table 5). The percentage of participants who had F or no grade in at least one subject (36.9%) was larger in comparison with in the *Complete mental health* group (15.5%), (Cohen’s h 0.50, p <0.001). The proportion of participants who had been a truant (51.0%) was larger compared with participants in the *Complete mental health* group (20.8%) and the *Vulnerable* group (27.5%) (Cohen’s h 0.64 and 0.49, respectively, p <0.001). Resilience scores were also lower in the current group compared to the *Complete mental health* and the *Vulnerable* group (Cohen’s d 1.39 and 0.69, respectively, p<0.001).

Further, a larger proportion of participants in the current group perceived feeling stressed to a considerable extent (76.1%) compared with participants in all the other three groups (21.9–53.3%, p<0.001) (Cohen’s h 0.48–1.15). The resilience and SSS scores in school were also lower among the *Troubled* participants compared with participants in another mental health status group (Cohen’s d 0.52–1.15).

### Associations between the background factors, school-related factors, stress, and resilience and mental health status group classification

The univariate regressions displayed that girls had elevated odds for being classified as *vulnerable* or having *Troubled* mental health status compared with boys (OR 1.95 and 5.02, respectively, p <0.001) (Table 6). Receiving grade F or no grade in at least one subject as well as being truant were factors associated with belonging to the S*ymptomatic but content* or *Troubled* mental health status groups (OR 5.68 and 3.17, respectively). Further, the odds ratios for being classified into the *Symptomatic but content*, *Vulnerable* and *Troubled* mental health status groups were higher among participants with a perception of feeling stressed to a considerable extent (OR 3.11–11.36) and those with lower resilience scores (OR 0.81–0.88).

**Table 6:** Unadjusted univariate binary logistic regressions exploring the odds of belonging to the different mental health status groups versus the *Complete mental health* status group. Significant associations are highlighted in bold.

	Outcome		
	Symptomatic but content (S)	Vulnerable (V)	Troubled (T)
	*(n*^a^ *= 708)*	*(n*^b^ *= 1533)*	*(n*^c^ *= 918)*
	OR (95% CI) *p-value*	OR (95% CI) *p-value*	OR (95% CI) *p-value*
Independent variables			
**Gender**			
Boy (ref)			
Girl	1.80 (0.98–3.29) *0*.*058*	**1.95 (1.59–2.40) *<0*.*001***	**5.02(3.64–6.93) *<0*.*001***
*Nagelkerke R* ^*2*^	*0*.*01*	*0*.*04*	*0*.*16*
**Country of birth**			
Sweden (ref)			
Other country	3.28 (1.44–7.50) *0*.*005*	1.16 (0.77-1-75) *0*.*466*	1.50 (0.88–2.56) *0*.*133*
*Nagelkerke R* ^*2*^	*0*.*02*	*<0*.*01*	*<0*.*01*
**Parents/legal guardians unemployed or on long-term sick-leave**			
No one (ref)			
At least one	2.55 (0.94–6.91) *0*.*066*	1.34 (0.86–2.12) *0*.*196*	1.83 (1.03–3.25) *0*.*039*
*Nagelkerke R* ^*2*^	*0*.*01*	*<0*.*01*	*0*.*01*
**Grade**			
Grade 7–9, lower secondary school (ref)			
Grade 10–12, upper secondary school	1.74 (0.95–3.19) *0*.*074*	1.36 (1.11–1.67) *0*.*003*	0.97 (0.72–1.30) *0*.*815*
*Nagelkerke R* ^*2*^	*0*.*01*	*0*.*01*	*<0*.*01*
**Given the grade F or no grade in any subjects**			
In no subjects (ref)			
In at least one subject	**5.68 (3.05–10.58) *0*.*001***	1.21 (0.92–1.58) *0*.*176*	**3.17 (2.28–4.42) *<0*.*001***
*Nagelkerke R* ^*2*^	*0*.*10*	*<0*.*01*	*0*.*07*
**Truancy**			
Never (ref)			
At some point during the semester or more often	**6.90 (3.64–13.06) <0.001**	1.44 (1.13–1.83) *0*.*003*	**3.96 (2.91–5.39) *<0*.*001***
*Nagelkerke R* ^*2*^	*0*.*13*	*0*.*01*	*0*.*12*
**Feeling stressed**			
No or to some extent (ref)			
Yes, to a considerable extent	**4.08 (2.21–7.54) *<0*.*001***	**3.11 (2.48–3.91) *<0*.*001***	**11.36 (8.07–15.99) *<0*.*001***
*Nagelkerke R* ^*2*^	*0*.*07*	*0*.*09*	*0*.*32*
**Resilience (CYRM-12)** ^d^	**0.88 (0.84–0.92) *<0*.*001***	**0.88 (0.86–0.89) *<0*.*001***	**0.81 (0.78–0.83) *<0*.*001***
*Nagelkerke R* ^*2*^	*0*.*12*	*0*.*16*	*0*.*40*
**Subjective social status (SSS) in school** ^d^ ^,^ ^e^	0.87 (0.69–1.09) *0*.*215*	**0.69 (0.64–0.74) *<0*.*001***	**0.55 (0.50–0.61) *<0*.*001***
*Nagelkerke R2*	*0*.*01*	*0*.*11*	*0*.*29*

^a^ Including participants classified as *Symptomatic but content* (n = 45) and with *Complete mental health* (n = 663).

^b^ Including participants classified as *Vulnerable* (n = 870) and with *Complete mental health (n = 663)*.

^c^ Including participants classified as *Troubled* (n = 255) and with *Complete mental health* (n = 663).

^d^ Continuous variables.

^e^ Since 200 of the 1,833 participants did not answer this question, n = 1633. The S and C groups compromised 589 participants. The V and C groups compromised 1,346 participants. The T and C groups compromised 784 participants.

Lower SSS in school scores were associated with higher odds of being classified in the *Vulnerable* and the *Troubled* groups (OR 0.55 and 0.69, respectively). The associations between gender, perceived stress, resilience, and SSS in school scores and being classified as belonging to the *Vulnerable* mental health status group remained significant in the multiple model (Table 7).

**Table 7:** Adjusted multiple binary logistic regression exploring the odds of belonging to the *Vulnerable* mental health status group versus the *Complete mental health* status group. Significant associations are highlighted in bold.

	*Outcome*
	**Vulnerable (V)**
	*(n*^a^ *= 1376)*
	OR (95% CI) *p-value*
*Independent variables*	
**Gender**	
Boy (ref)	
Girl	**1.88 (1.44–2.46)) *<0*.*001***
**Birth country**	
Sweden (ref)	
Other country	0.92 (0.55–1.54) *0*.*762*
**Parents/legal guardians unemployed or on long-term sick-leave**	
No one (ref)	
At least one^c^	1.00 (0.59–1.69) *0*.*993*
**Grade**	
Grade 7–9, lower secondary school (ref)	
Grade 10–12, upper secondary school	1.41 (1.10–1.82) *0*.*008*
**Given the grade F or no grade in any subjects**	
In no subjects (ref)	
In at least one subject	0.75 (0.52–1.07) *0*.*112*
**Truancy**	
Never (ref)	
At some point during the semester or more often	1.16 (0.85–1.56) 0.352
**Feeling stressed**	
No or to some extent (ref)	
Yes, to a considerable extent	**2.23 (1.70–2.94) *<0*.*001***
**Resilience (CYRM-12)** ^b^	**0.87 (0.85–0.89) *<0*.*001***
**Subjective social status in school (SSS)** ^b^	**0.76 (0.70–0.82) *<0*.*001***
**Nagelkerke R** ^**2**^	0.30
**Hosmer and Lemeshow test**	
χ 2 (df) *p-value*	5.10 (8) *0*.*747*

^a^ Including participants without missing data on SSS in school scores, classified as *Vulnerable* (n = 788) and with *Complete mental health (n = 558)*.

## Discussion

This study aimed to I) describe four mental health groups according to the dual-factor model in a Swedish community population of adolescents and II) examine the association between mental health status subgroups and background factors, school-related factors, stress, and resilience in this population.

### Proportions of adolescents in the four mental health status groups

The results revealed that about a third of the adolescents had *Complete mental health* status (36.2%). Participants with *Vulnerable* mental health status comprised the largest group (47.5%). Notably, this group would have been classified as mentally healthy in the traditional model of assessing mental health, merely addressing the presence/absence of mental health problems. More than one out of ten adolescents (13.9%) belonged to the *Troubled* mental health status group, while only a small proportion (2.5%) was classified as belonging to the *Symptomatic but content* group. These findings differ from previous research, which report that more than half of the adolescents (57–78%) belong to the *Complete mental health* status group [9, 15, 17–22, 24, 26, 28]. Furthermore, in these studies, the proportions of *Vulnerable* youth are reported to be smaller than in the current study, namely 5–20% versus 47.5% in the current study.

Comparisons of results can however be misleading since the instruments for measuring mental well-being and mental health problems differ between studies as well as methods used for dichotomization of the respective scales. For instance, in the aforementioned studies, mental well-being was measured by questions merely assessing emotional well-being [13, 21], and not comprising the psychological or social functioning, as defined by Keyes [37] and the WHO [12, 14]. The results might also be influenced by contextual factors [28], since most studies on the topic have been conducted in the United States [9, 15, 17–20, 22–24, 26]. Moreover, in the current study, the larger proportion of *Vulnerable* adolescents (48%) compared to previous studies may reflect a decrease in adolescents’ mental well-being, since a majority of the latter studies were conducted over ten years ago.

In the current study, a smaller proportion of participants were classified into the *Symptomatic but content* mental health status group than in previous studies. Here too, comparisons with other studies are challenging due to different measures and the other aforementioned factors. Our findings on the proportion of adolescents with *Troubled* mental health status (13.9%) are in line with some previous studies, reporting that approximately 15–17% of the adolescents are classified according to this group [15, 22, 24, 26, 27]. However, none of these studies used the SDQ to measure mental health problems.

Overall, the current study provides novel information on the distribution between the four groups, which is important to consider when planning for interventions to improve adolescents’ mental health. The results highlight the importance of supportive interventions geared toward the large proportion of adolescents with low mental well-being (with *Vulnerable* or *Troubled* mental health status) to achieve better emotional well-being as well as psychological and social functioning. Such multicomponent interventions strengthening *Vulnerable* adolescents’ mental well-being could protect this large group from future mental health problems and physical illness [10, 37, 56–58]. As for *Troubled* adolescents, increased mental well-being might support recovery from their mental health problems [10]. Schools provide an important arena, where programs to promote well-being and prevent mental health problems can be implemented [1]. Further research on adolescents’ mental health status according to the dual-factor model would be useful to explore comparisons among different countries, instruments, and methodologies. Preferably, such research should use reliable and valid instruments, capturing both emotional well-being and psychological and social functioning, according to the three-fold definition of mental well-being [13], as well as mental health problems.

### Background factors, school-related factors, stress, and resilience in relation to mental health status

The results demonstrated differences in background factors, school-related factors, stress, and resilience between the four mental health status groups. Since two-thirds of the adolescents did not experience complete mental health, these findings are worth considering when planning for interventions to improve adolescents’ mental health.

In line with previous research [17, 18, 24, 26], differences in subject grades and truancy were evident between participants with *Complete mental health* and participants with *Symptomatic but content* and *Troubled* mental health status, with the largest effect size between the *Complete mental health* status group and the *Symptomatic but content* mental health status group. The results imply that interventions are needed to support school achievements and reduce school truancy among adolescents with mental health problems, regardless of their level of mental wellbeing.

Adolescents with *Complete mental health* were more resilient and less likely to have a high stress level compared with adolescents in the other three mental health status groups, with the largest effect sizes in comparison with the *Troubled* mental health status group.

This is coherent with existing literature suggesting that resilience and low stress are associated with high mental well-being and absence of mental health problems [9, 22, 32]. However, Lyons and colleagues [9] reported that high stress is associated with *Troubled* mental health status and not with *Vulnerable* or *Symptomatic but content* mental health status, as in the current study. This might be explained by different measures of stress between the studies.

While Lyons et al. [9]measured stress as the experience of stressful life events (like parent’s loss of job) over the last year, the current study comprised the broader question “Do you feel stressed?” The latter possibly captured other kinds of stress, e.g., associated with decreased mental well-being.

Nevertheless, in the current study, feeling stressed was associated with higher odds ratios for being classified as having *Troubled* mental health status than as having *Symptomatic but content* or *Vulnerable* mental health status. Still, the results highlight the importance of preventing high stress levels and promoting resilience for all adolescents who do not have complete mental health. Here, school-based programs may be useful, as well as interventions that affect social determinants of health, for instance, parental support [1, 59].

The results also disclosed gender differences, i.e., girls were more likely to have *Vulnerable* or *Troubled* mental health status compared with boys, with the highest odds for the latter mental health status. As far as we are aware, no previous study has reported a relation between gender and mental health status classification [9, 15, 17, 20, 24]. The gender differences evident in our study, however, correspond to the literature suggesting that mental health problems are more common among girls than among boys [1, 4]. The current study’s results also support the assumption that low mental well-being is more common among girls than among boys [1, 35], thus adding knowledge to the limited amount of research in this area. Previous literature has suggested that gender differences in adolescents’ mental health are influenced by societal gender norms, placing women and girls lower than men and boys in the societal hierarchy, for instance, causing girls to perceive pressure to perform well in school [34, 60].

Therefore, interventions to reduce traditional stereotypic gender norms may be useful to increase the proportion of girls with complete mental health status. However, these interventions may also be useful in promoting complete mental health among boys [1, 16, 34]. Further research exploring whether the association between stress and mental health status is moderated by resilience would also be useful in supporting the delivery of interventions. Previous research has reported that adolescents living in families with high socioeconomic status are more likely to have *Complete mental health* than belonging to the *Troubled* group [15, 21, 22, 24]. In the current study, no differences were found between the mental health status groups, when SES was explored by parent/legal guardians’ occupation. However, SSS in school scores, which also might be used as an indicator of SES [52], differed between the mental health status groups. For instance, participants with *Complete mental health* rated themselves higher on SSS in school, compared with participants in the *Vulnerable* and *Troubled* mental health status groups, with the largest effect size between the *Complete mental health* and *Troubled* groups.

This finding suggests that SSS in school is related to both low mental well-being and mental health problems. Overall, more research is needed regarding the association between SSS in school and mental health status.

### Strengths and limitations

The LHU survey used for data collection comprised 59 questions, with a total of 143 items, only available in Swedish. Thus, the magnitude and language could be considered as a limitation, since it could have affected non-native speaking adolescents’ ability to complete the survey. Students with disabilities attending a special school and students in upper secondary school who attended language introduction programs were not included in the study, which may affect the generalizability of the results. Moreover, participants with other gender identity than a boy or a girl were excluded, due to the limited numbers. In addition, the survey did not reach adolescents who had finished compulsory secondary school and who were not participating in upper secondary school studies. There is also a possibility that students with a high tendency of being truant or inability to attend school due to mental health problems were absent from school on the day their class responded to the survey. This could possibly have affected the study results regarding, e.g., proportions of students in the four mental health status groups. However, generalizability was strengthened by participation from all lower and upper secondary schools in the surveyed region.

In line with all surveys, participants possibly providing socially desirable answers or not answering entirely truthfully could be a limitation. Still, the exclusion of outliers based on length and weight was an effort to eliminate respondents with unreliable answers. Also, Bonferroni correction with adjusted p-value reduced the risk for type one errors due to multiple testing.

The study was strengthened by the use of the survey Life and Health of Youth (LHY), which previously has been used in other Swedish regions. During the planning of the LHY, comments on the questionnaire were obtained from 32 adolescents aged 15–18 years and some adjustments were made to avoid information bias. Further, the MHC-SF and the SDQ measures, included in the survey, have demonstrated psychometric properties in previous research [42, 44, 48–51, 61] and displayed acceptable to excellent internal consistency in the current study.

However, both the MHC-SF and the SDQ are self-reported instruments and no additional clinical mental health assessments were conducted to confirm our findings.

The study was conducted with a total sample of all students in grades 9–12 on Gotland, which is a strength. However, the response rate was 62% and despite the fact that 1,833 participants were included in the study, the sample size could be considered too small for some analyses. For instance, there could be a risk of type two error for results regarding birth country and parent/legal guardians’ occupation in relation to mental health status, since the smallest mental health status group, *Symptomatic but content*, comprised only 45 participants. Moreover, the same group, alongside the *Troubled* group were considered as too small to be used as outcome variables in multivariate analyses. Thus, multivariable regression was only conducted exploring associations between factors and the *Vulnerable* group. Still, the results from the univariate analyses exploring the odds ratios for classification into the *Vulnerable group* remained significant in the adjusted multivariable model.

As in all cross-sectional studies, the results in the current study only give information on the association between different factors and the four mental health status groups, and not causality. The current study, however, was the first attempt to investigate the dual-factor model in a Swedish community population of students in grades 7–12, in upper secondary school, involving a measure of mental well-being according to the three-fold definition of mental well-being. The results contribute to knowledge on associations between different factors and being classified into the four mental health status groups, which can be used in planning for interventions aiming to reduce health inequities in adolescents’ mental health. However, when evaluating the results, it is necessary to take into account that Gotland is a small region and thus the generalizability to urban settings and other countries may be limited.

## Conclusions

Our findings suggest that using the dual-factor model in measuring adolescents’ mental health adds valuable information, compared with using only traditional methods to measure the presence or absence of mental health problem. The largest group in the current study, the *Vulnerable* group, might have been classified as mentally healthy due to the absence of mental health problems and therefore be overlooked if a traditional way of measuring adolescents’ mental health was used. It is important to acknowledge the mental health status of the *Vulnerable* group, since differences in characteristics are evident between this group and adolescents with *Complete mental health*. The results suggest that interventions to strengthen mental well-being constitute an important complement to prevention of mental health problems in order to achieve better mental health among adolescents. Further, differences in participants’ characteristics between the four mental health status groups reveal a potential in providing interventions targeting resilience, stress, and stereotypic gender norms to support adolescents without complete mental health to achieve better mental health.
